# Analysis of C3 Gene Variants in Patients With Idiopathic Recurrent Spontaneous Pregnancy Loss

**DOI:** 10.3389/fimmu.2018.01813

**Published:** 2018-08-07

**Authors:** Frida C. Mohlin, Piet Gros, Eric Mercier, Jean-Christophe Raymond Gris, Anna M. Blom

**Affiliations:** ^1^Department of Translational Medicine, Lund University, Malmö, Sweden; ^2^Crystal and Structural Chemistry, Bijvoet Center for Biomolecular Research, Department of Chemistry, Utrecht University, Utrecht, Netherlands; ^3^Laboratory of Hematology, University Hospital, Nimes, France

**Keywords:** reproductive immunology, miscarriage, complement system, C3, mutation

## Abstract

Miscarriage is the most common complication of pregnancy. Approximately 1% of couples trying to conceive will experience recurrent miscarriages, defined as three or more consecutive pregnancy losses and many of these cases remain idiopathic. Complement is implicated both in the physiology and pathology of pregnancy. Therefore, we hypothesized that alterations in the C3 gene could potentially predispose to this disorder. We performed full Sanger sequencing of all exons of C3, in 192 childless women, with at least two miscarriages and without any known risk factors. All exons carrying non-synonymous alterations found in the patients were then sequenced in a control group of 192 women. None of the identified alterations were significantly associated with the disorder. Thirteen identified non-synonymous alterations (R102G, K155Q, L302P, P314L, Y325H, V326A, S327P, V330I, K633R, R735W, R1591G, G1606D, and S1619R) were expressed recombinantly, upon which C3 expression and secretion were determined. The L302P and S327P were not secreted from the cells, likely due to misfolding and intracellular degradation. Y325H, V326A, V3301I, R1591G, and G1606D yielded approximately half C3 concentration in the cell media compared with wild type (WT). We analyzed the hemolytic activity of the secreted C3 variants by reconstituting C3-depleted serum. In this assay, R1591G had impaired hemolytic activity while majority of remaining mutants instead had increased activity. R1591G also yielded more factor B activation in solution compared with WT. R1591G and G1606D showed impaired degradation by factor I, irrespectively if factor H, CD46, or C4b-binding protein were used as cofactors. These two C3 mutants showed impaired binding of the cofactors and/or factor I. Taken together, several alterations in C3 were identified and some of these affected the secretion and/or the function of the protein, which might contribute to the disorder but the degree of association must be evaluated in larger cohorts.

## Introduction

Spontaneous pregnancy loss, or miscarriage, is the most common complication of pregnancy and includes all pregnancy losses from conception until 24 weeks of gestation. It is believed that as many as 50% of all conceptions and 15% of clinically recognized pregnancies end up in miscarriage (1). The definition of recurrent spontaneous pregnancy loss (RSPL) differs among international societies. The American Society for Reproductive Medicine defines RSPL as two or more pregnancy losses (2), affecting around 5% of couples trying to conceive, while The European Society for Human Reproduction and Embryology defines it as three or more consecutive losses (3), affecting around 1% of couples trying to conceive. Over the years, various etiologies have been identified, such as genetic, structural, autoimmune, endocrine, thrombophilic abnormalities, together with infections. However, approximately 40–50% of the cases still remain idiopathic (1).

Pregnancy is a complex process, where on the one hand significant adaptations of the immune system is needed to tolerate the semiallogenic fetus but on the other hand, an active immune system is needed to defend the mother and fetus from infections. The complement system, which is a pivotal part of our innate immunity, has been shown to play an important role for a successful pregnancy. Studies have shown that too little or too much complement at the wrong time during pregnancy can have devastating consequences for the pregnancy outcome. Too much complement activation in the placenta may lead to placental damage, with a potential risk of fetal loss and therefore complement inhibition in the placenta is of great importance. Complement inhibitors, such as CD46, CD55, and CD59, are expressed in the placenta and on the surface of the trophoblast (4–6), and the critical role of these inhibitors is demonstrated by the embryonic lethality in mice deficient in Crry (7), an important mouse complement inhibitor that resembles human CD46. Previously, we hypothesized that alterations in the complement inhibitors C4b-binding protein (C4BP), CD46, and CD55 might be associated with an increased complement activation, at the trophoblastic maternal interface, leading to pregnancy loss. Several alterations in C4BP and CD46 were identified and some of these caused altered expression and/or function of the proteins (8). However, complement is not only needed for fighting infections during pregnancy but also for proper placental and fetal development. Despite the complement inhibitors presence in the placenta, some degree of complement activation is seen during normal pregnancy. One example of this importance is that mice deficient in C1q, had abnormal development of the placenta, reduced litter size and fetal weight (9). These mice demonstrated a preeclamptic-like phenotype with proteinuria and increased blood pressure, which indicates that C1q plays an important role for a successful pregnancy. Furthermore, C1q in human placenta has also been implicated in preeclampsia (6).

Complement component C3 is the key protein of the complement system and composed of two polypeptide chains, α- and β-chain. The importance of C3 is demonstrated by the high plasma concentration of 1–1.5 mg/ml (10). The cleavage of C3 by C3-convertases is the central reaction of all three complement pathways and results in the biologically active components C3a, an anaphylatoxin and C3b, a potent opsonin. C3b has an exposed thioester bond, allowing C3b to rapidly bind covalently to cell surfaces and promote further complement activation. C3b will participate to form the C5-convertase and in the final step of the complement cascade, the membrane-attack complex is formed, which has the ability to lyse some target cells, e.g., Gram-negative bacteria (11). Activation of complement can be limited by degradation and inactivation of C3b by factor I, in the presence of different cofactors, such as factor H, CD46, and C4BP (12). C3, along with C3b and most importantly iC3b (the inactivated form of C3b), has been shown to be embryotrophic factors. These factors are mainly derived from the uterus and promote embryonic growth prior to the development of the placenta. In humans, the most abundant embryotrophic factor is embryotrophic factor 3 and this was later found to contain mainly C3, C3b, and iC3b (13). It has also been demonstrated that rat C3 on the visceral yolk sac is important for early embryonic development and in explant rat embryo culture, adding intact C3 significantly favors development, without the requirement of C3 activation (14). C3 knock-out mice also demonstrate smaller blastocysts, higher resorption rates, and smaller sizes of the placenta (15). In humans, C3 levels was shown to be higher in patients suffering three consecutive miscarriages, compared with women who went on having a live birth after two miscarriages (16). *In vitro* data also suggest that mutations in *FOXD1*, a transcription factor that directly seems to target C3, leading to both decreased and increased C3 expression, were deleterious (17). Genetic variants in the *C3* gene have also been reported to be associated with severe preeclampsia (18). Thus, C3 appears to have an important physiological role in the early phase of pregnancy and in placental development and a subtle fine-tuning of the C3 level is necessary for optimal function.

Due to the role of C3 in the physiology and pathology of pregnancy, we hypothesized that maternal mutations and polymorphisms in C3 might be associated with pregnancy loss. Herein, we performed full Sanger sequencing of all coding exons of *C3* in women experiencing idiopathic RSPL and we found several heterozygous non-synonymous alterations. The C3 mutants were expressed recombinantly and some of the alterations affected the secretion and/or the function of the protein, which might contribute to the disorder.

## Materials and Methods

### Patients and Controls

Our cohort was previously described in detail (8). Briefly, patients with recurrent pregnancy losses were referred to the Department of Gynecology and Obstetrics or the Hematology Laboratory, University Hospital of Nîmes, France. In total, 1,359 women were pre-selected but the focus was on the 962 most severe cases, defined by at least three consecutive embryonic losses before the 10th gestational week, or two consecutive fetal losses at and beyond the 10th gestational week, all occurring in childless women. These women were screened for classical risk factors for pregnancy loss, such as abnormal parental karyotypes, infectious diseases during pregnancy, uterine anatomical abnormalities, diabetes mellitus, thyroid dysfunction, hyperprolactinaemia prior to luteal phase defects, erythroblastosis fetalis, immune thrombocytopenic purpura, feto-maternal alloimmune thrombocytopenia, and antiphospholipid antibodies. Women were excluded from the study if they had any of the above classical risk factors, previous occurrence of superficial or deep vein thrombosis, were positive for constitutional thrombophilia, preeclampsia, or were of non-Caucasian origin, since this might have introduced consistent confounding heterogeneities in the local frequencies of the polymorphisms and mutations under focus. In the end, 453 women fulfilled all of the criteria and after informed consent was obtained, 429 patients were finally recruited. DNA samples from controls were obtained from women referred to the Department of Gynecology and Obstetrics for a systematic medical exam such as implementation of a new contraception or evaluation of the pelvic floor after pregnancy. Women with no previous pregnancy loss but with at least two uneventful pregnancies were selected (*n* = 261) and similar to cases, these controls were also screened for classical risk factors for pregnancy loss. In total, 224 controls were finally recruited, after informed consent was obtained.

### DNA Sequencing

All exons of C3 were sequenced in 192 patients by Polymorphic DNA Technologies (Alameda, CA, USA). A nested PCR amplification of the specific exons preceded the Sanger dideoxy sequencing. The primers used are listed in Table 1. The identified non-synonymous alterations found in the patients were then analyzed in a control group of 192 women. All patients and controls were randomly chosen from the cohort defined above. There were no statistical differences in baseline characteristics (age, BMI, ethnicity, and risk factors for vascular diseases) between the patients and controls (Table 2). The NHLBI Exome Sequencing Project database^1^ was used for investigating the frequencies of the identified C3 non-synonymous alterations in the general population. The PolyPhen software^2^ was used for determining the potential effect on the protein function. The sequencing data have been deposited at DDBJ/EMBL/GenBank Targeted Locus Study project under the accession KCDD00000000. The version described in this paper is the first version, KCDD0100000.

**Table 1:** Primers used for sequencing of all C3 exons.^a^

Exon no.	Outer primers 5′	Outer primers 3′	Inner primers 5′	Inner primers 3′
1	TGCTGGGGAACATGC	ACAGGCAGCCCAAATAT	GGACTGAAAAGCTTAGGA	ATCATTCCCAACCTGCTAA
2	CCAGAGAAGATAATGGCAT	AAGGAGAGGCGACTC	CGGCAGTCACTGGAT	GGCTTAGAAAGGGAGAA
3–4 (a)	CTCGCACCTCCTTCA	TGACCATGACCGTCC	TCCCTCCCCAAAACG	GTGAAGATCCGATAGAGAA
3–4 (b)	AGGGAGTTCAAGTCAGA	CTGTGTCTCTGCCACT	GTTCGTGACCGTGCA	GCCTCTCCTAAGCCT
5–7 (a)	CTGGGTCCCTGTTCTTA	TCGAAACTGGGCAGC	CTTAACCACAGTAGACACT	GAGATGGCGTTGGTG
5–7 (b)	ACCAGCTTGGCGTCT	CCCACCTGGTCTTCA	CTTGTCTTGGGACATTC	CCTCACCTGGCTCTT
8–9 (a)	TGGGAGTGGGGCAA	GGTGGCAGACACGTA	AGAGGCCAGCAGTG	CAGGTCTTCTGCTCG
8–9 (b)	TCCCGTAGGTTCCTC	CCATCTTCATACTCGGC	GAGGGAACTGCCTTTG	GAACCCCACTTTCACTC
10–11 (a)	CAAGGCCAGTATAAAGGT	TTGGCCACGCCATCT	GTATGAAAGTGGACTCTACT	GACTGCACAGTGTCCT
10–11 (b)	AGATCCACTTCACCAAG	CGCAGGAGAGAACCT	AAGACACCCAAGTACTTC	CTATGCAGATGAGAATATCTGT
12	ATCTCCAGATCCCTAACT	TCCCGCCTTCAACAG	CTAACTTAATCCCATCTGCAA	ACAGGCTGGCATCAG
13	CGCTACTACACCTACC	ACAGTTGAGAGACAGAGA	CCACCTGGAAACCTC	GAGAGAGGAGTAGGGA
14	CTCCCCCCAACCTT	GTCCAACCTCATTCCCA	TTCTGTCTTTCCACTCTAG	CAGCTTCAACCATCCCT
15	ACAGAGCAAGACTCCAT	GTGTTCCTGCTCCCAT	AGGAAGGGCAGAAGC	TGATGGAAGAGCAAACTGA
16	ATGCTGGGAGCAGAG	CAGCACTTGCGCAG	GCCTGGGTTCAAGC	TACTTGCCGACTGCG
17	CGTGCAGGAGCCA	GGCCTCCTCCAGC	AGGGAGGAGGGGA	CCACCCCCACCAG
18	GTCCTGACCTCAAGC	GACAGGCTGGAAGG	CACCCACCTCAGC	GCCTCCATGTAGGTC
19	CATGCACCTGCTATCTC	TGACTCTTGGCTGAATTCT	TCTGGAATCACAGTGCA	AGGAACTAAGGATTCCCA
20	TCATTTGTCGCCATTGAC	GCACGATGACATATGGA	TTCACAGGCTTCAGCAA	CTGGTGACGCCTCTT
21	CCGAGCCGTTCTCTA	ACTTTACCCCAATCCTG	GGCAGAACCAAGAGCT	CAAATTTCCTAAGCTGGACA
22–23	CTCCCAAAGTGCTAGGA	GTAAAAGAGGGAAAACAGATC	GCCCGGCAATGCTA	GGGCTGTTCTAGCTGAA
24	CCGCAGTCACATCC	GGCCTCAAATGAGGG	CTCGCCTGTCCCTA	GGCTAGGAGAGAAGGT
25	CCCTTGTCCTCTTGAGA	CTGGTTTGCCTTCCCA	TCCACCTCCTCGTTC	GTGCTTAAGGATGCTTAATG
26	GCATCCTTAAGCACGGA	ATGCTGGAGTGACGC	CAACAGCTGGGTCCT	CCCAATCTTTGCAAAGG
27	CATTGACATTTGCAGAAGGA	CATGTGAAACTATTAGAGGG	CAAATTGTTGCATGAATTAATACTA	TGTATTAGATGAGTTAAGTGCT
28	GCTAAAATATCTGGGAAATTCT	ACCTGCGAAAACTTAGAG	GAAATTCTCAAAAATGGACAAATCT	CCAGCTTGATACCTTAG
29	AAACAAGAGAGGTAAGGCA	AACCTATAGATGGGTTTGC	CAAGTAATTGAGAGCCTC	TTCTCAACTCCACTGCAAT
30	CCTCAGAGTCCAGTGA	TGGGTTTCTGTTCCTTAC	CCCTGCACTCTCTAG	AACCCTCATTTTCCTTGGT
31–32	GGTACAGTCACCCAG	GGTGACCTAGCTTCTTA	GCTGTCTCCCCCTA	CCTGAGGCCTCTTTTCT
33	GCTGACATTGTGTCTCC	GTGTACTTTCCTCCATTGAT	CCAAAGATCAACTCACC	TTACGTGGTCATTGCTG
34–35	CTTTGGGGAAATGTTTCCTAAA	ATAGAAGATGCTGGGTTGA	CCTCTGTGCTGCTATG	CCTTATTTTACAAGCTCCTTC
36	GCCACACCTAATTGAAAGA	AGAACCTCAGAACCTCAA	AGAAAGGTAGTCTCACTG	CGTGGGACCTTCATAC
37–38 (a)	CCAAAGATCAGAAAGTAGAG	TGGCTCACAGGCCTT	GAGGGGTTGAAGACCTA	CTTCCAGGGTGACCTT
37–38 (b)	GGAGGATGGAAAGCTGA	GCCCCTCACAATACATG	CTCTGCCGTGATGAAC	CACACACCACAGTCAC
39–40	GGCCATAGTGTGACTG	CTTCCCGATGATGTAGC	GCTGTGATTCTGCAACTT	GAATGGCAGGTCAGGAA
41	TTCATCAGCCCCATCAA	CACTAGCAGGCGAAC	CTCATGTGGGGTCTC	ATGCGGTGGGAACAG

*^a^The specific exons were first amplified from genomic DNA using nested PCR. The nested PCR product was subsequently sequenced, using Sanger dideoxy method and the inner primer pairs*.

**Table 2:** Baseline characteristics of the sequenced patients and controls.^a^

	Patient group	Control group
*N*	192	192
Age, years^b^	29 (4) [18–44]	30 (5) [17–44]
Age >35 years^c^	8 (4.2%)	10 (5.2%)
BMI, kg/m^2b^	25.9 (4.2) [13.5–34.1]	25.6 (4.5) [15.3–36.1]
BMI >30^c^	21 (10.9%)	19 (9.9%)
BMI <18.5^c^	2 (1.1%)	3 (1.6%)
**Ethnicity^c^**
Caucasian-European	158 (82.3%)	155 (80.7%)
Caucasian-North African	24 (12.5%)	26 (13.5%)
Black African	7 (3.6%)	9 (4.7%)
Asian	3 (1.6%)	2 (1.1%)
**Risk factors for vascular diseases^c^**
Current smokers	22 (11.5%)	20 (10.4%)
Hypertension	7 (3.6%)	5 (2.6%)
Hypercholesterolemia	9 (4.7%)	10 (5.2%)
Hypertriglyceridemia	7 (3.6%)	8 (4.2%)
Diabetes mellitus	2 (1.9%)	3 (1.6%)

*^a^No statistical differences were detected between the two groups, for either of the parameters. The significance was assessed with Mann-Whitney test for the quantitative data and Chi-Square test for the qualitative data*.

*^b^Quantitative data: median, interquartile range, and range values*.

*^c^Qualitative data: numbers and percentages*.

### Proteins

Factor B, factor D, factor H, C3, and properdin were purchased from Complement Technology. Factor I (19), C4BP (20), CD55 (21), and CD46 (21) were expressed recombinantly and purified, as described previously.

### Expression of C3 Variants

The non-synonymous C3 variants of interest (Table 3) were expressed recombinantly. cDNA coding for human C3 was used as template and the mutations were introduced by site directed mutagenesis, according to the manufacturer’s instruction (QuikChange Lightning, Agilent Technologies). The primers used for mutagenesis are listed in Table 4. Automated DNA sequencing confirmed the correct mutations and the constructs were cloned into the mammalian expression vector pCEP4 (Thermo Fisher Scientific). The constructs were transiently transfected into either human embryonic kidney-293 adherent cells (ATCC) using Lipofectamine 2000 (Thermo Fisher Scientific) or Freestyle 293-F cells (Thermo Fisher Scientific) using FreeStyle MAX reagent (Thermo Fisher Scientific). To evaluate the expression and secretion levels of the mutants, the C3 concentration in the supernatants and cell lysates after transient transfection in adherent cells, were determined by ELISA (described below). The same volume (5 µl) of these cell supernatants was also analyzed by Western Blotting, both under non-reducing and reducing (25 mM DTT) conditions. A polyclonal goat anti-human C3 antibody (Quidel), followed by a HRP-conjugated rabbit anti-goat antibody (Dako), were used for detection. The blots were developed using enhanced chemiluminescence (Merck-Millipore). The supernatants from the Freestyle 293-F cells were collected, concentrated, C3 concentration determined by the same ELISA and used in the functional assays. Supernatant from cells transfected with empty pCEP vector were treated in the same manner and used as negative control in all assays. For some functional assays, C3b-like molecules were needed, and this was achieved by treatment of the C3 cell media with 0.1 M methylamine hydrochloride pH 8.0, for 1 h at 37°C, followed by a buffer exchange to TBS (Zeba Spin desalting columns, Thermo Fisher Scientific). Methylamine will create C3met, a C3b-like molecule, by breaking the labile thioester bond in C3 and inducing a conformational change.

**Table 3:** Mutations and polymorphisms found in *C3*.^a^

Amino acid^b^	cDNA ATG+1	SNP no.	Exon	Domain	Polyphen^c^	MAF, %^d^	Frequency in patients, % (no.)	Frequency in controls, % (no.)
p.R102G	c.304 C>G	rs2230199	3	β-chain/MG1	Benign	15.5	40.1 (77)	32.8 (63)
p.K155Q	c.463A>C	rs147859257	4	β-chain/MG2	Benign	0.3	0.5 (1)	
p.S297SfsX5	c.891delG	Novel	9	β-chain/MG3	–	0	1 (2)	
p.L302P	c.905 T>C	Novel	9	β-chain/MG3	Probably damaging	0	0.5 (1)	
p.P314L	c.941 C>T	rs1047286	9	β-chain/MG3	Benign	14.9	34.4 (66)	25.5 (49)
p.Y325H	c.973 T>C	Novel	9	β-chain/MG3	Possibly damaging	0	0.5 (1)	
p.V326A	c.977 T>C	Novel	9	β-chain/MG3	Possibly damaging	0		0.5 (1)
p.S327P	c. 979T>C	Novel	9	β-chain/MG3	Probably damaging	0		0.5 (1)
p.V330I	c. 988G>A	Novel	9	β-chain/MG3	Probably damaging	0		0.5 (1)
p.K633R	c.1898 A>G	rs140655115	15	β-chain/LNK	Benign	0.07	1 (2)	
p.R735W	c.2203 C>T	rs117793540	17	α-chain/ANA/C3a	Probably damaging	0.3	0.5 (1)	0.5 (1)
p.R1591G	c.4771 A>G	Novel	40	α-chain/C345C	Probably damaging	0	0.5 (1)	
p.G1606D	c.4817 G>A	Novel	40	α-chain/C345C	Probably damaging	0	0.5 (1)	
p.S1619R	c.4855 A>C	rs2230210	41	α-chain/C345C	Possibly damaging	0.2	0.5 (1)	0.5 (1)

*^a^All mutations were found in heterozygous form. None of the found alterations were significantly associated with the disorder, as determined by Fisher’s exact test*.

*^b^Numbering including signal peptide, Met = 1*.

*^c^Polyphen predication of the protein function: http://genetics.bwh.harvard.edu/pph2/*.

*^d^MAF, minor allelle frequency in NHLBI Exome Sequencing Project database*.

**Table 4:** Primer sequences (5′–3′) used to introduce site directed mutations in *C3*.

C3 mutation	Primer sequence (5′–3′)^a^
R102G	GTCAGAAAAGGGGGGCAACAAGTTCGTG
K155Q	CACCGTCAACCACCAGCTGCTACCC
L302P	GGGAGGTTGTGCCGAGCCGGAAGGTAC
P314L	GGTGCAGAACCTCCGAGCAGAAGAC
Y325H	GGGAAGTCTTTGCACGTGTCTGCCAC
V326A	GGAAGTCTTTGTACGCGTCTGCCACCGTC
S327P	GTCTTTGTACGTGCCTGCCACCGTCATC
V330I	CGTGTCTGCCACCATCATCTTGCACTC
K633R	CGGGCAGTGGGAGGGATTACGCCGG
R735W	CATCACAGAGCTGTGGCGGCAGCACGC
R1591G	CCCATCAAGTGCGGAGAAGCCCTGAAGC
G1606D	CTACCTCATGTGGGATCTCTCCTCCG
S1619R	GAGAAGCCCAACCTCCGCTACATCATCGG

*^a^Nucleotides corresponding to the changed amino acid residue are underlined*.

### C3 Concentration Determination by ELISA

To determine the C3 concentration after protein expression, an ELISA was performed. Two polyclonal antibodies were used, to eliminate the risk that a monoclonal antibody will recognize the mutants differently. Microtiter plates were coated with rabbit anti-human C3c antibody (Dako), diluted in 75 mM sodium carbonate, pH 9.6. The wells were washed with washing buffer (50 mM Tris–HCl, pH 8.0, 0.15 M NaCl, 0.1% Tween) and blocked with 3% fish gelatin (Norland Products) in washing buffer. The samples were diluted in blocking buffer, supplemented with 10 mM EDTA, and incubated on the plate for 1 h at 37°C. Purified C3 from Complement Technology was used as a standard. C3 was then detected using a goat anti-human C3 antibody (Quidel), followed by a HRP-conjugated rabbit anti-goat antibody (Dako). After 1 h incubation at RT for each of the antibodies, the plates were developed using OPD-tablets (Kem-En-Tec). The absorbance at 490 nm was measured spectrophotometrically.

### Hemolytic Assay

To assess the hemolytic activity of the C3 variants, a hemolytic assay using C3-depleted serum was performed, essentially as described previously (22). Briefly, antibody-sensitized sheep erythrocytes (Håtunalab AB) was incubated in a V-bottom microtiter plate with 1% C3-depleted serum (Quidel) and four different concentrations (0–2 µg/ml) of either wild type (WT) or mutant C3, in DGVB++ buffer (2.5 mM veronal buffer pH 7.3, containing 70 mM NaCl, 140 mM dextrose, 0.1% porcine gelatin, 1 mM MgCl_2_, and 0.25 mM CaCl_2_). The plate was incubated for 1 h at 37°C, during shaking, followed by centrifugation to pellet unlysed erythrocytes. The hemolytic activity was then evaluated by measuring the absorbance, i.e., amount of released hemoglobin, in the supernatant at 405 nm.

### Degradation of C3 by Factor I

C3b can be degraded by factor I, in the presence of cofactors such as factor H, CD46, and C4BP. C3 cell media treated with methylamine (C3met) were used. In a total volume of 15 µl, 20 ng of C3met, 25 ng factor I, and either 200 ng factor H or 100 ng CD46 were added in TBS. For C4BP, 20 ng C3met, 50 ng factor I, and 4 µg C4BP were mixed. The samples were then incubated for 60 and 90 min, and the reaction was terminated by addition of SDS-PAGE sample buffer, containing 25 mM DTT. The samples were heated at 95°C for 5 min, applied on a 10% SDS-PAGE, and transferred to a PVDF-membrane for Western Blotting. The C3 Western Blot procedure is described above. The intensity of the intact α′-chain of C3met and the 46 kDa degradation product were analyzed, and the results are shown as a ratio of the two.

### Direct Binding Assays

Binding of C3 to the different ligands factor H, CD46, C4BP, factor I, CD55, factor B, and properdin was assessed in plate assays. The ligands were immobilized on microtiter plates at 5 µg/ml (except factor B which was coated at 10 µg/ml) in PBS and the plates were washed and blocked as described above for the C3 ELISA. The different C3 cell media (mock cell medium was used as negative control), treated with methylamine, were then diluted to four different concentrations in a low salt buffer (10 mM Tris, pH 7.2, 25 mM NaCl, 0.05% Tween 20, 4% BSA) and incubated on the plate for 2 h at 37°C. For binding to factor H and CD46, C3 variants were tested at 250, 125, 62, and 31 ng/ml; for C4BP, properdin and CD55 at 500, 250, 125, and 62 ng/ml; for factor I at 1,000, 500, 250, and 125 ng/ml; and for factor B at 2,000, 1,000, 500, and 250 ng/ml. The amount of bound C3met was then detected using a rabbit anti-human C3c antibody (Dako), followed by a HRP-conjugated goat anti-rabbit antibody (Dako). Both antibodies were incubated for 1 h at 37°C and developed as described above.

### Factor B Cleavage

The cleavage of factor B by factor D was determined in solution, in the presence of methylamine-treated C3 WT, R1591G, or mock cell medium as negative control. A mixture of 2 µg/ml factor B, 2 µg/ml C3, and 0.1 µg/ml factor D was prepared in Mg^2+^ EGTA buffer (2.5 mM veronal buffer, pH 7.3, 70 mM NaCl, 140 mM glucose, 0.1% gelatin, 7 mM MgCl_2_, and 10 mM EGTA) and incubated for 0.5, 1, 2, and 4 h at 37°C. At each time point, 10 µl was removed from the mixture, and the reaction stopped by addition of SDS-PAGE sample buffer. The samples were heated at 95°C for 5 min, subjected to SDS-PAGE (10% gel, non-reduced), and transferred to a PVDF-membrane for Western Blotting. Intact factor B, together with the cleavage products Bb (60 kDa) and Ba (33 kDa), were then detected using a polyclonal goat anti-human factor B antibody (Complement Technologies), followed by a HRP-conjugated rabbit anti-goat antibody (Dako). Both antibodies were diluted 1:10,000 and developed using enhanced chemiluminescence (Merck-Millipore). The intensity of the intact factor B band and Bb degradation product were analyzed, and the results are shown as a ratio of the two.

### Structural Analysis

Structure files were downloaded from the Protein Data Bank (23) and visualized using the molecular graphics program PyMOL.^3^

### Statistical Analysis

Fisher’s exact test was used to assess statistical association between the found C3 alterations and the disorder. The statistical differences in baseline characteristics between the patient and the control group were assessed with Mann–Whitney test for the quantitative data and Chi-square test for the qualitative data. For all experimental data in the figures, statistical significance was determined using either one-way or two-way ANOVA with Dunnett’s multiple comparison test. The results are shown as the mean + SD of at least three independent experiments and values of *p* < 0.05 were considered significant (*p < 0.05, **p < 0.01, and ***p < 0.001).

## Results

### Alterations Identified in *C3*

All exons of *C3* were sequenced in 192 patients with RSPL. The exons carrying identified non-synonymous alterations found in the patient group were then analyzed in a control group of 192 women. Altogether, 13 heterozygous, non-synonymous alterations were found both in patient and control groups (Table 3). Seven novel alterations in C3 were found in total, four in the patient group (L302P, Y325H, R1591G, and G1606D) and three in the control group (V326A, S327P, and V330I). The remaining alterations have been identified previously in other patient cohorts or are common polymorphisms (R102G and P314L). We also identified a novel heterozygous deletion (p.S297SfsX5) in two patients, which results in a frame shift and an introduction of a premature stop codon. This mutant was not expressed recombinantly as it could only yield a 30-kDa truncated fragment of C3 without function. None of the found alterations in the patient group were statistically significantly associated with RPSL, as determined by Fisher’s exact test. With the available sample size we had a power of at least 0.80 to detect an association for alleles with risk allele frequencies >0.07, assuming a population prevalence of 0.05, an alpha of 0.05, and a genotype relative risk of 2. Power was calculated using the Genetic Power Calculator (24).

### Expression, Secretion, and Characterization of C3 Variants

**Figure 1:**
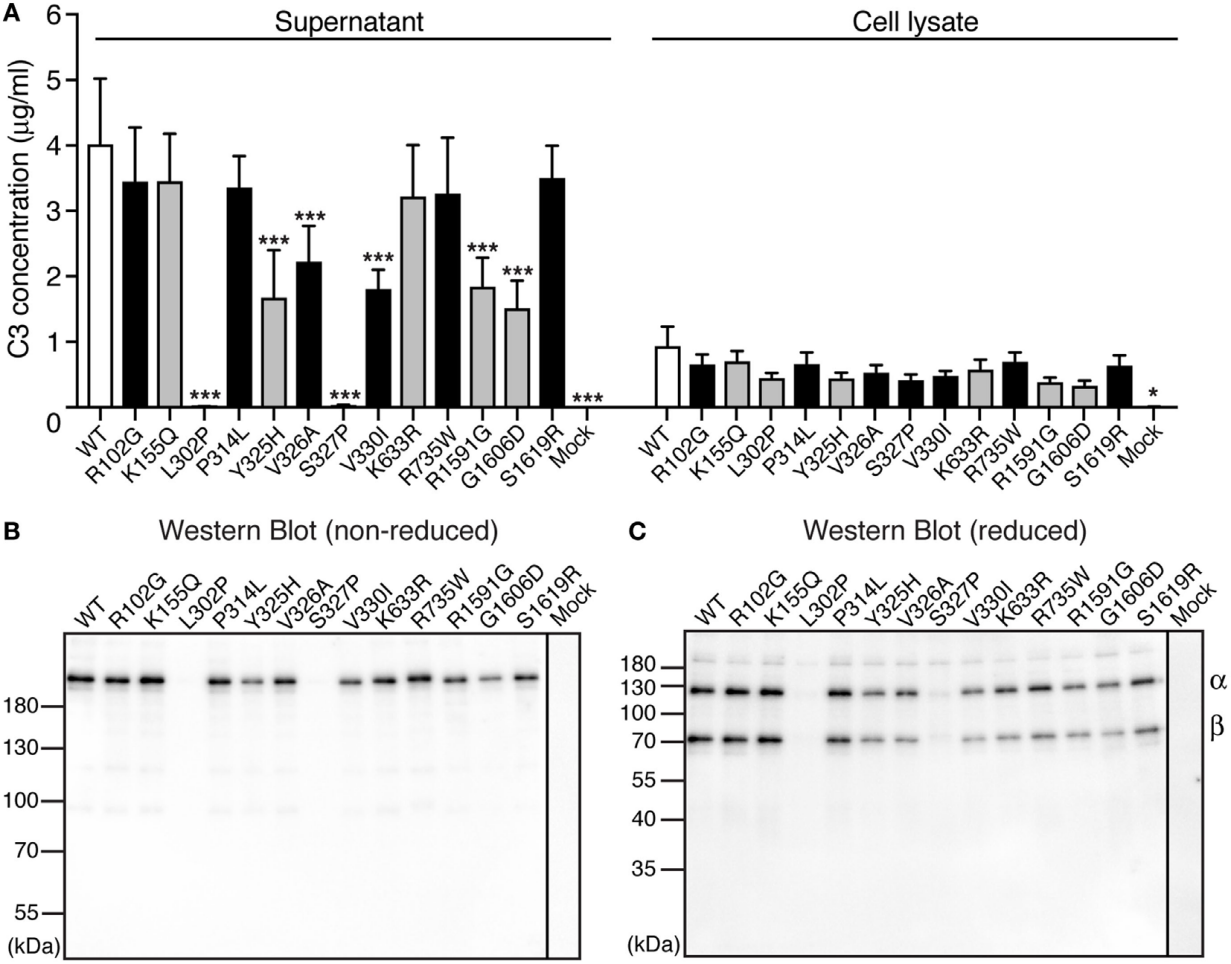
Expression of C3 variants. **(A)** C3 variants, together with wild type (WT) and negative control (mock = empty vector), were transiently transfected into adherent human embryonic kidney (HEK) 293 cells. After four independent transfections, the C3 concentration was measured both in the conditioned cell media (supernatant) and the cell lysate using ELISA. The results are shown as mean + SD and the statistical significance of the differences between WT and mutants was determined using two-way ANOVA with Dunnett’s multiple comparisons test, *p < 0.05, ***p < 0.001. The C3 alterations identified only in patients are marked in gray. **(B,C)** After transient transfections, the same volume of each cell supernatant was also subjected to SDS-PAGE under both **(B)** non-reducing and **(C)** reducing conditions, followed by Western blotting. C3 was detected using a polyclonal anti-C3 antibody.

To be able to elucidate whether the found C3 variants altered the expression, secretion, or function of the protein, all 13 identified alterations were expressed recombinantly. Transient transfections revealed that two of the mutants (L302P, identified in one patient and S327P, identified in one control) were expressed but not secreted from the cells into the supernatant (Figure 1A). Five other alterations (Y325H, V326A, V330I, R1591G, and G1606D) yielded approximately half C3 concentration in the supernatant compared with WT. The supernatants from the transient transfections were also subjected to Western Blotting under both non-reducing (Figure 1B) and reducing (Figure 1C) conditions. All mutants migrated with the same apparent velocity to 185 kDa for non-reduced C3 and to 110 and 75 kDa for the α- and β-chain, respectively, under reducing conditions.

### Hemolytic Activity

**Figure 2:**
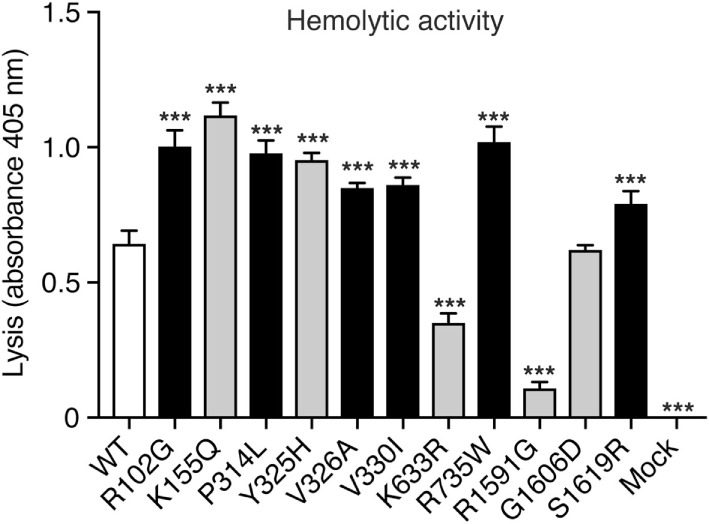
Hemolytic activity. C3-depleted serum was reconstituted with either C3 wild type (WT), the different C3 variants or the negative control (corresponding volumes of cell media from mock-transfected cells). This was added to antibody-sensitized sheep erythrocytes and the hemolytic activity, i.e., lysis of the erythrocytes, was determined by measuring the absorbance of the supernatant at 405 nm (free hemoglobin). C3 was added in different concentrations but for clarity only one concentration (2 µg/ml) is shown in the figure. The experiment was performed three independent times in duplicates, and the results are shown as mean + SD. Statistical significance of the differences between WT and mutants was determined using one-way ANOVA with Dunnett’s multiple comparisons test, ***p < 0.001. The C3 alterations identified only in patients are marked in gray.

To assess the hemolytic activity of the C3 variants, C3-depleted serum was reconstituted with C3 WT or variants (or corresponding volumes of mock medium as negative control), added to antibody-sensitized sheep erythrocytes and the amount of hemoglobin released from lysed erythrocytes was measured spectrophotometrically. Four concentrations of C3 were tested (2, 1, 0.5, and 0.25 µg/ml) in the hemolytic assay but for clarity only the result for 2 µg/ml is shown in Figure 2. Several variants showed increased hemolytic activity compared with WT while K633R and R1591G showed instead impaired activity. However, for the K633R mutant, the impaired activity was only clearly observed for the one shown concentration. The other three tested concentrations showed the same hemolytic activity as for WT. The rest of the mutants displayed the same activity for all tested concentrations (data not shown).

### Factor B Cleavage in Solution for R1591G

**Figure 3:**
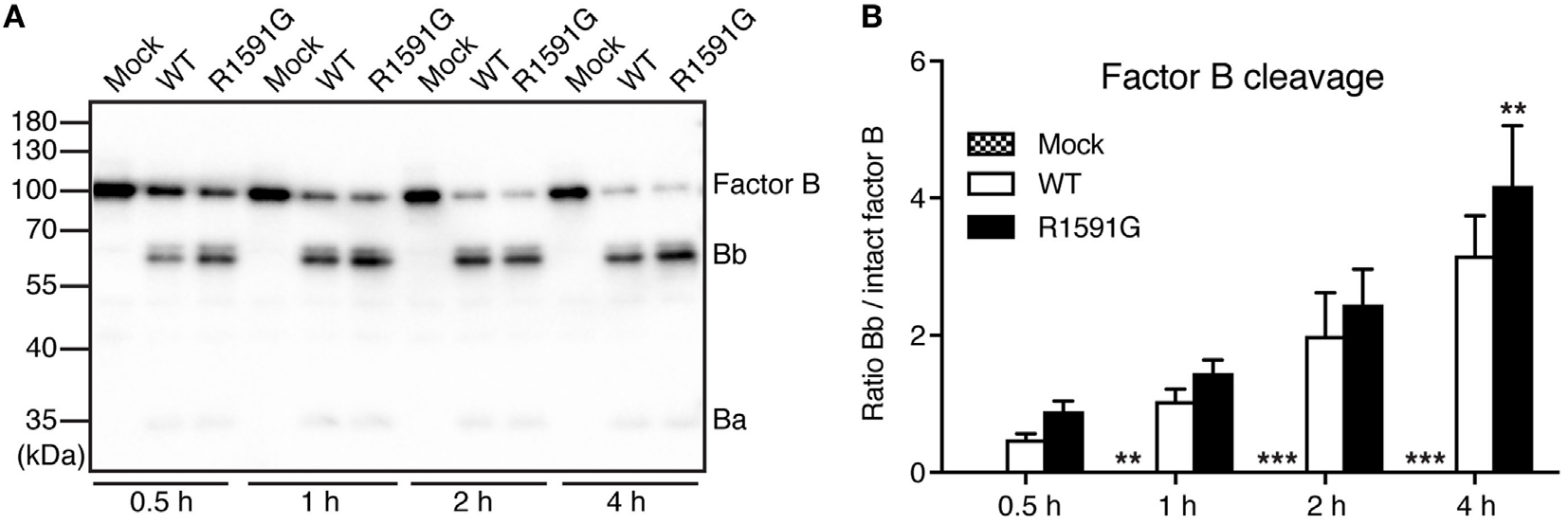
Factor B cleavage in solution. Methylamine-treated C3 wild type (WT) or R1591G (or mock medium as negative control) was incubated with factor B and factor D for indicated time points, before being subjected to Western Blotting and factor B detection. A representative blot is shown in panel **(A)**. The bands corresponding to intact factor B as well as the Bb degradation product were quantified by densitometry. Data in panel **(B)** show the ratio between the intensity of these two bands. Each sample was analyzed in a single replicate, and the data are shown as mean + SD from four independent experiments. Two-way ANOVA with Dunnett’s multiple comparisons test was used to calculate statistical significance of the difference between WT and R1591G, **p < 0.01 and ***p < 0.001.

Since R1591G showed decreased hemolytic activity, we hypothesized that this mutant might cause more cleavage of factor B by factor D in solution and thereby less C3 will be available for the cell surface. Indeed, in the presence of R1591G, an increased cleavage of factor B in solution was observed, compared with C3 WT (Figure 3). This was statistically significant after 4 h cleavage but the trend was visible at all tested time points.

### Degradation of C3 by Factor I

**Figure 4:**
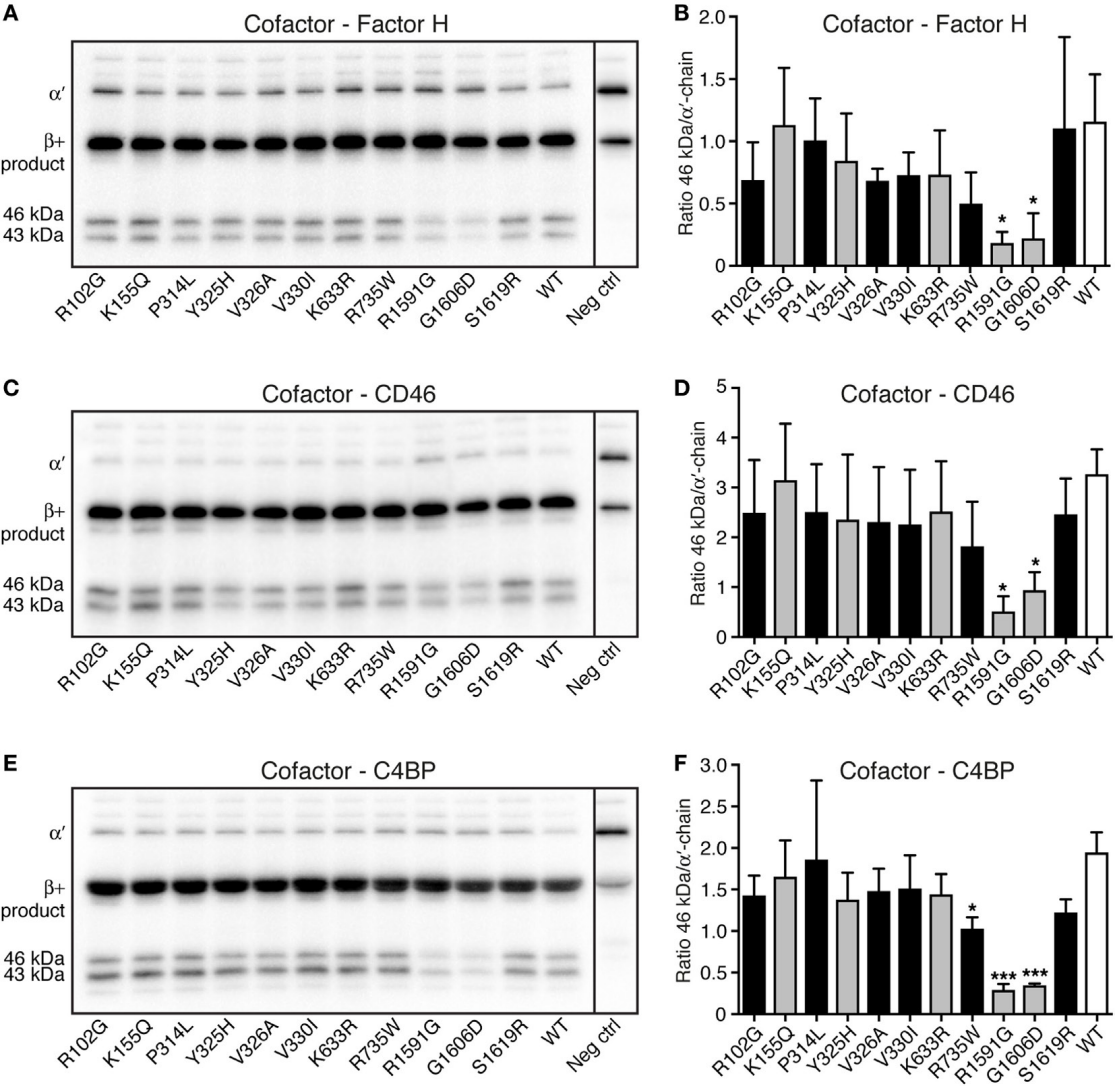
Degradation of C3met by factor I, in the presence of different cofactors. The ability of the C3 variants to be degraded by factor I, in the presence of the different cofactors **(A,B)** factor H, **(C,D)** CD46, and **(E,F)** C4b-binding protein (C4BP) was determined. C3met, either wild type (WT) or variants, was incubated with factor I, together with the cofactor for 90 min. The degradation of C3met was evaluated by Western Blotting [representative blots can be seen in panels **(A,C,E)**] and the bands corresponding to intact α′-band of C3met as well as the 46 kDa degradation product were quantified by densitometry. Data in panels **(B,D,F)** show the ratio between the intensity of these two bands. The negative control shown in the blots is C3 WT at 0 min. Each sample was analyzed in a single replicate, and the data are shown as mean + SD from at least three independent experiments. Statistical significance of the differences between WT and mutants was determined using one-way ANOVA with Dunnett’s multiple comparisons test, *p < 0.05 and ***p < 0.001. The C3 alterations identified only in patients are marked in gray.

Degradation assays in fluid phase were performed to study if the different C3 variants could be degraded by factor I, in the presence of the cofactors factor H, CD46, and C4BP. C3 cell media were treated with methylamine to create C3met, a C3b-like molecule, and this was incubated for either 30 min (data not shown) or 90 min (Figure 4) with factor I and the different cofactors. Two of the mutants (R1591G and G1606D) showed statistically significantly impaired degradation compared with WT, regardless of which cofactor used. Degradation assays using factor H (Figures 4A,B), CD46 (Figures 4C,D), and C4BP (Figures 4E,F) all displayed the same result. The same trend was also seen at 30 min cleavage (data not shown).

### Binding Assays

**Figure 5:**
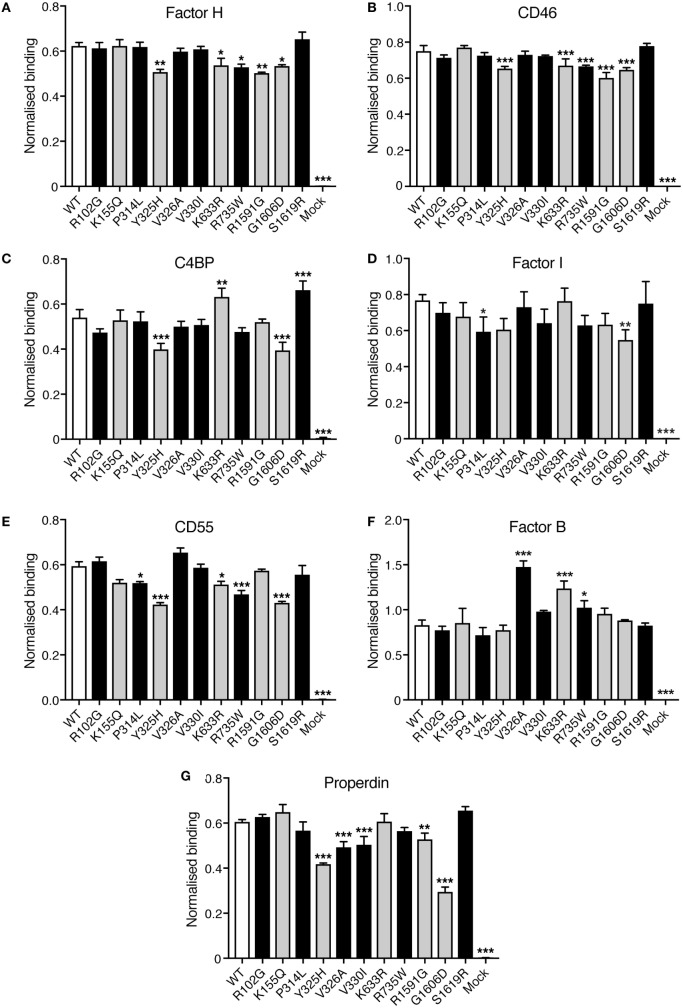
Binding of factor H, CD46, C4b-binding protein (C4BP), factor I, CD55, factor B, and properdin to C3met. Microtiter plates were coated with the different ligands for C3b: **(A)** factor H, **(B)** CD46, **(C)** C4BP, **(D)** factor I, **(E)** CD55, **(F)** factor B, and **(G)** properdin. C3met, either WT, variants or corresponding volumes of cell media from mock-transfected cells as a negative control, was added to the coated plates and the ability of the variants to bind the different ligands was detected using a polyclonal anti-human C3 antibody. Four different concentrations of C3met were tested but for clarity only one concentration for each ligand is shown in the figure (125 ng/ml for factor H and CD46; 250 ng/ml for C4BP, properdin, and CD55; 1 µg/ml for factor B; and 500 ng/ml for factor I). Each concentration was analyzed in duplicates, and the data are shown as mean + SD from three independent experiments. One-way ANOVA with Dunnett’s multiple comparisons test was used for determining the statistical significance of the differences between WT and mutants, *p < 0.05, **p < 0.01, and ***p < 0.001. The C3 alterations identified only in patients are marked in gray.

Direct binding assays were performed to elucidate if the C3 variants showed altered binding to factor H, CD46, C4BP, factor I, CD55, factor, and properdin. Corresponding volumes of mock medium was used as negative control. Four different concentrations of C3 were tested in each binding assay but for clarity only one concentration is shown in Figure 5. Small differences in binding could be observed. R1591G, that showed both impaired hemolytic activity and impaired degradation by factor I, also showed a somewhat decreased binding to factor H, CD46, and properdin. The other mutant, G1606D, that also showed impaired degradation by factor I, also displayed somewhat impaired binding to factor H, CD46, C4BP, factor I, CD55, and properdin.

### Structural Analysis

Next, we tried to rationalize the functional consequences of the identified and studied alterations in the context of the known C3 (pdb-id: 2A73) (25) and C3b (pdb-id: 5FO7) (26) structures.

**Figure 6:**
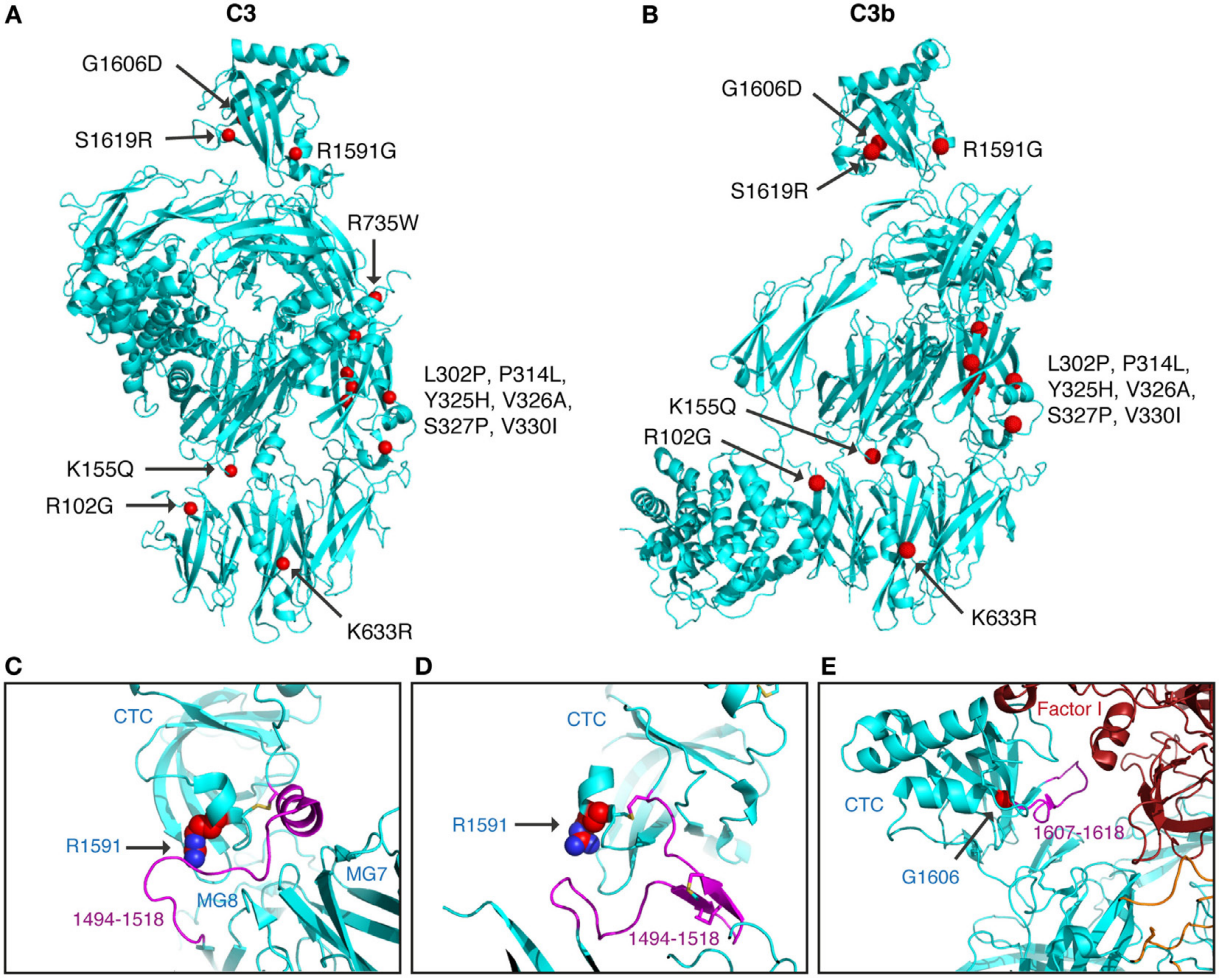
Structural analysis of C3 alterations. The C3 alterations analyzed in this study were mapped onto the **(A)** C3 (pdb-id: 2A73) and **(B)** C3b (pdb-id: 5FO7) structures, using the molecular graphics program PyMOL. R735W is located in C3a and therefore absent in the C3b molecule. For R1591G; structures are shown of **(C)** C3 (pdb-id: 2A73) and **(D)** C3b (pdb-id: 5FO7). Both C3 and C3b are colored in cyan with indicated in purple the neck region (res. 1494–1518), connecting the CTC domains to the C3 and C3b body. R1591 is indicated by spheres and disulfide bridge Cys1518–Cys1590 shown in sticks. **(E)** For G1606D; the structure of C3b—mini-factor H—factor I (pdb-id: 5O32) is shown. The glycine C[alpha] atom is indicated by a red sphere. Mutation G1606D likely induces local rearrangement affecting its subsequent loop (res. 1617–1618, highlighted in purple) that is directly involved in contacting factor I.

All C3 alterations analyzed in this study were mapped onto both the C3 (Figure 6A) and the C3b structure (Figure 6B). R102G is the common polymorphism also known as C3 S/F (slow/fast) and located in the macroglobulin (MG) 1 domain. Mutants K155Q, located in the MG2 domain; K633R in the LNK domain; R735W in ANA/C3a domain and S1619R in the C345C (CTC) domain, all showed normal expression and normal or only slightly altered function.

Mutants L302P, P314L, Y325H, V326A, S327P, and V330I are all located in the MG3 domain of C3. For two of the mutants, L302P and S327P, C3 was not secreted from the cells after transient transfections. In these mutants, leucine 302 and serine 327 are changed to proline residues, which most probably will cause misfolding and degradation of the protein intracellularly. The others are relatively structurally subtle mutations, changing a proline into leucine, a tyrosine into histidine, a valine into an alanine, and a valine into a leucine in MG3. Structurally, MG3 plays a critical role in the conversion from C3 to C3b and its contacts with MG7 and MG8 are changed drastically when converting C3 into C3b by removal of the anaphylatoxin (ANA/C3a) domain. Apparently, the minor alterations in the MG3 domain affected the secretion of the protein (all except P314L showed decreased secretion) and also resulted in an increased hemolytic activity.

Mutant R1591G showed impaired hemolytic activity but instead yielded increased cleavage of factor B in the solution. In C3, R1591 is involved in interactions with residues of the “neck” region (res. 1494–1517) in native C3. Mutation R1591G possibly destabilizes these interactions and thereby alters or destabilizes the MG7-MG8 domain arrangement (Figures 6C,D). Destabilization of the MG7-MG8 arrangement in C3 is likely to induce generation of C3(H_2_O) more readily, leaving less native C3 available for hemolytic activity. Moreover, enhanced generation of C3(H_2_O) also explains the observed increased cleavage of factor B, due to soluble C3 convertase, i.e., C3(H_2_O)Bb, generation.

Both R1591G and G1606D showed impaired degradation by factor I, independently of which cofactor that was used. Both mutations R1591 and G1606 are located in the C-terminal CTC domain. Interactions between the C3b-CTC domain and the heavy chain of factor I (pdb-id: 5O32) are needed to bind factor I and activate its catalytic serine–protease domain for cleaving C3b (26). Mutation G1606D possibly induces local rearrangements that disturb neighboring contact sites required for factor I binding (Figure 6E). R1591G, however, is positioned opposite of the factor I binding interface on the CTC domain. Putatively, a distortion of the “neck” conformation mis-orients the CTC domain for proper factor I binding needed for inducing catalytic activity.

## Discussion

Miscarriage is unfortunately a common complication of pregnancy and RSPL affects around 1–5% of couples trying to conceive. Evidence-based treatments, e.g., aspirin and heparin treatment for antiphospholipid syndrome and surgical corrections for uterine abnormalities, have improved the outcome for couples over the years but 40–50% of the cases still remain idiopathic. The complement system has been shown to be involved both in the physiology and the pathology of pregnancy. Furthermore, C3—the central protein of the complement cascade—appears to have an important role in the early phase of pregnancy and in the development of the placenta. In this study, we therefore set out to determine whether maternal variations in C3 are associated with idiopathic RSPL.

We performed full Sanger sequencing of all exons of the *C3* gene, in 192 patients with RSPL. The exons carrying identified alterations were then analyzed in a control group of 192 women and a total of 13 heterozygous non-synonymous alterations were found in both groups. Four of these (L302P, Y325H, R1591G, and G1606D) were novel mutations only found in the patient group and three (V326A, S327P, and V330I) were novel ones only found in the control group. We found the two common C3 polymorphisms in many of both the patients and controls; R102G (27) and P314L (28), which both have been associated with age-related macular degeneration (AMD) in many studies (29–31). R102G is also known as the C3S/F (slow/fast) allele and has been associated not only with AMD but also other immune-mediated disorders, such as IgA nephropathy, systemic vasculitis, partial lipodystrophy, and membranoproliferative glomerulonephritis type II (32). K155Q is a rare mutation, found previously in AMD patients (33) and we found this mutation in one RSPL patient as well. We also identified K633R in two RSPL patients, which has previously been identified to be a rare variant in atypical hemolytic uremic syndrome patients (34). Both R735W and S1619R were each found in one patient and one control and the former has previously been reported before in atypical hemolytic uremic syndrome (35) and the latter in AMD (36). None of the C3 alterations identified in the patients were associated with RSPL. However, we used a relatively small cohort and several of the found mutations are rare variants, which makes it difficult to appropriately statistically analyze the association between rare variants and disease. To be able to conclude a significant association, replication in larger cohorts is needed.

We decided to recombinantly express and analyze all our found alterations, also those only found in the control group. The women in the control group were selected on the basis of no previous pregnancy loss, at least two uneventful pregnancies and no known classical risk factors for pregnancy loss. However, they could potentially have other, unknown medical conditions, not related to pregnancy loss. Transient transfections of all C3 variants revealed that two mutants, L302P (identified in one patient) and S327P (identified in one control), were not secreted from the cells. These proline substitutions will most probably misfold the protein, leading to intracellular degradation. However, it should be noted that all identified C3 alterations in this study were in heterozygous form, which means that the individuals with the L302P and S327P alterations could theoretically have C3 concentration in blood decreased by 50%. We could unfortunately not confirm this, due to lack of blood samples from these individuals. Functional testing of the mutants that were secreted revealed that several of the alterations had increased hemolytic activity compared with C3 WT, e.g., R102G and P314L, which is in accordance with previous reports (37, 38). However, in that previous report, the authors also observed a 1.3-fold lower binding affinity for R102G to factor H, something we did not observe. The reason for this could be the use of different methods; we detected factor H binding using a microtiter plate assay and the former report used surface plasmon resonance. The K155Q mutant was previously reported in AMD (39) with impaired degradation of this mutant by factor I, in the presence of factor H as a cofactor. When using CD46 as a cofactor, the activity was normal. However, in our degradation assay, this mutant showed normal activity, for both the cofactors factor H and CD46. Furthermore, we did not see any altered binding for K155Q to factor H in our microtiter plate assay, which is in accordance with Seddon et al. However, Seddon et al. did see a decreased binding of K155Q to factor H using surface plasmon resonance. The only difference we observed for this mutant was an increased hemolytic activity. R735W was previously tested functionally, for its binding to CD46, factor H, factor B, and soluble CR1, together with its degradation by factor I, using CD46 as the cofactor (35). In that report, no functional changes were observed. In our report, we could see a slightly impaired degradation by factor I, when using C4BP as a cofactor but in accordance with the previous report, the activity was normal when using CD46 as the cofactor. We could also see a somewhat decreased binding to factor H and CD46, together with an increased binding to factor B. However, these changes were only minor.

Two novel mutations, importantly only found in the patient group, displayed the most functional impairment and also decreased secretion of the protein into the cell media after transient transfection: R1591G and G1606D. Both of these mutations showed impaired degradation by factor I, independently of which cofactor that was used (factor H, CD46, or C4BP). Binding of R1591G to factor H and CD46 was slightly impaired but we could not observe any impaired binding to factor I in a microtiter plate assay. However, to become active, factor I needs to bind to C3b in a way that it stabilizes the conformation of its catalytic domain. R1591G is positioned opposite of the factor I binding interface on the CTC domain. A distortion of the “neck” conformation could mis-orient the CTC domain for proper factor I binding needed for inducing catalytic activity. R1591G also displayed a decreased hemolytic activity but instead an increased cleavage of factor B in the solution. This might lead to consumption of C3 in the solution, resulting in less activity on the cell surface. Structural analysis of this mutant indicated that R1591G is involved in interactions with the neck region of native C3 and could possibly destabilize that region. This could also lead to more C3(H_2_O) generation and subsequently less native C3 available for hemolytic activity. The other mutant, G1606D, also showed impaired degradation by factor I. Structural analysis of this mutant revealed that this amino acid change could induce a significant local rearrangement, which might affect the residues that are involved in binding to factor I. We did indeed observe a slightly impaired binding of this mutant to factor I, together with impaired binding to factor H, CD46 and C4BP, which could all together result in the impaired degradation we see by factor I.

Taken together, several C3 alterations were identified both in patients and controls. Since we used a relatively small cohort, none of the found alterations in the patient group were associated with RSPL in a statistically significant manner. To be able to determine an association with the disease, larger cohorts need to be analyzed. Two mutants (L302P, found in one patient and S327P, found in one control) were not secreted from the cells, probably due to misfolding and two others (R1591G and G1606D), found in patients, showed both decreased secretion and functional impairment. Some of the identified C3 alterations might result in *in vivo* consequences and contribute to RPSL.

## Ethics Statement

This study was carried out in accordance with the recommendations and approval by the University Hospital of Nîmes Institutional Review Board and ethics committee (ref# 2001-12-07). All subjects gave written informed consent in accordance with the Declaration of Helsinki.

## Author Contributions

AB and J-CG designed the study. J-CG and EM provided the patient and control samples, together with the clinical data. FM performed the functional experiments. PG performed the structural analysis. FM, AB, PG, and J-CG wrote the manuscript, and all authors approved it.

## Conflict of Interest Statement

The authors declare that the research was conducted in the absence of any commercial or financial relationships that could be construed as a potential conflict of interest.

## Footnotes

^1^http://evs.gs.washington.edu/EVS (Accessed: 20 February, 2018).

^2^http://genetics.bwh.harvard.edu/pph2/ (Accessed: 20 February, 2018).

^3^https://pymol.org/2/ (Accessed: 2 March, 2018).

## Abbreviations

AMD, age-related macular degeneration; C4BP, C4b-binding protein; HEK, human embryonic kidney; RSPL, recurrent spontaneous pregnancy loss; WT, wild type.
